# Increasing Fosfomycin Resistance in *Klebsiella pneumoniae* Isolates Causing Bloodstream Infections in Hospitalized Adults: Seven Years of Surveillance

**DOI:** 10.3390/antibiotics15100991

**Published:** 2026-10-07

**Authors:** Nazmiye Ülkü Tüzemen, İzzet Burçin Saticioglu, Muhammed Duman, Uğur Önal, Seçil Ak Aksoy, Sezen Yusuf, Egon A. Ozer, Halis Akalın, Cüneyt Özakın

**Affiliations:** 1 Department of Medical Microbiology, Faculty of Medicine, Bursa Uludag University, 16059 Bursa, Türkiye; 2 Department of Aquatic Animal Diseases, Faculty of Veterinary Medicine, Bursa Uludag University, 16059 Bursa, Türkiye; iburcinsat@uludag.edu.tr (İ.B.S.); mduman@uludag.edu.tr (M.D.); 3 Department of Infectious Diseases and Clinical Microbiology, Faculty of Medicine, Bursa Uludag University, 16059 Bursa, Türkiyehalis@uludag.edu.tr (H.A.); 4 Division of Infectious Diseases, Northwestern University Feinberg School of Medicine, Chicago, IL 60611, USA; 5 Center for Pathogen Genomics and Microbial Evolution, Havey Institute for Global Health, Northwestern University, Chicago, IL 60611, USA

**Keywords:** fosfomycin, *Klebsiella pneumoniae*, fosA, fosA2, fosA3, fosA4, fosA5, sodium phosphonoformate

## Abstract

Introduction: We investigated seven-year temporal trends in fosfomycin resistance among *Klebsiella pneumoniae* bloodstream isolates and characterized resistant isolates phenotypically and molecularly. Methods: We included 198 non-duplicate bloodstream isolates collected from hospitalized adults during 2017–2023. Fosfomycin MICs were determined by agar dilution and evaluated using the 2023 EUCAST intravenous clinical breakpoint, FDA susceptibility test interpretive criteria (STIC), and current EUCAST (T)ECOFF. Temporal trends were assessed by logistic regression. Phenotypic evidence of FosA-like enzyme production was assessed by PPF disk testing, acquired *fosA* variants by PCR and Sanger sequencing, and two representative isolates by Oxford Nanopore WGS. Results: Using the 2023 EUCAST breakpoint, 25.3% of isolates were resistant, with a significant annual increase in resistance odds (OR 1.22, 95% CI 1.04–1.44; *p* = 0.019). Using FDA STIC, 12.6% were resistant, with a significant temporal increase (OR 1.43, 95% CI 1.13–1.81; *p* = 0.003). According to the EUCAST (T)ECOFF, 6.1% were non-WT, with a non-significant trend (OR 1.36, 95% CI 0.99–1.86; *p* = 0.057). Among 47 viable resistant isolates, 36.2% were PPF-positive. No acquired *fosA*, *fosA2*, *fosA3*, or *fosA4* genes were detected. Published *fosA4* primers showed non-specific amplification. WGS identified chromosomal *fosA5* in both sequenced isolates. Conclusions: Fosfomycin resistance increased significantly according to the 2023 EUCAST breakpoint and FDA STIC, whereas the ECOFF-based non-WT trend was not significant. The absence of common acquired *fosA* variants and non-specificity of published *fosA4* primers highlight the need for careful molecular validation.

## 1. Introduction

*Klebsiella pneumoniae* is one of the leading causes of healthcare-associated bloodstream infections, particularly among hospitalized patients with severe underlying illnesses and Intensive Care Unit (ICU) admission. The global emergence of multidrug-resistant (MDR) and carbapenem-resistant *K. pneumoniae* (CRKP) has substantially limited treatment options and is associated with increased mortality, prolonged hospitalization, and healthcare costs [1]. As resistance to last-line agents continues to increase, optimizing the use of available antibiotics has become an important component of antimicrobial stewardship programmes.

Fosfomycin, an old antibiotic with broad activity against Gram-negative bacteria, has regained clinical interest because of its activity against MDR Enterobacterales and its favorable pharmacokinetic profile [2,3,4]. Oral fosfomycin formulations are widely used, particularly for the treatment of uncomplicated urinary tract infections. Increasing fosfomycin use, together with co-selection phenomena, may contribute to the development and dissemination of fosfomycin resistance among Enterobacterales [5]. Intravenous fosfomycin has increasingly been used in combination regimens for severe infections, including bloodstream infections caused by carbapenem-resistant organisms [2,3,4]. Following regulatory approval, intravenous fosfomycin was introduced into clinical practice in our hospital in October 2018.

The increasing use of fosfomycin raises concerns regarding the emergence of resistance. Fosfomycin resistance in *K. pneumoniae* can arise through several mechanisms, including alterations in the MurA target, impaired fosfomycin uptake through the GlpT and UhpT transport systems and their regulatory pathways, and enzymatic inactivation by Fos enzymes [2,5]. Alterations involving *glpT*, *uhpT*, and regulatory genes such as *uhpA*, *uhpB*, *cyaA*, *ptsI*, and *crp* may therefore contribute to reduced fosfomycin susceptibility [2,5]. In addition, *K. pneumoniae* commonly carries chromosomal *fosA* homologues, whereas acquired *fosA* variants represent potentially transferable resistance determinants of epidemiological relevance [5,6,7]. Testing for fosfomycin susceptibility in non-*Escherichia coli* Enterobacterales remains technically challenging. The Clinical and Laboratory Standards Institute (CLSI) and the European Committee on Antimicrobial Susceptibility Testing (EUCAST) recommend the agar dilution (AD) method as the reference standard for fosfomycin susceptibility testing; however, this method is labor-intensive and is not routinely implemented in many clinical microbiology laboratories [4]. Because laboratory characterization of fosfomycin resistance in *K. pneumoniae* remains challenging, phenotypic methods, including sodium phosphonoformate (PPF)-based FosA inhibition assays, together with molecular approaches, have been used to further investigate resistant isolates [8]. Continuous local surveillance of fosfomycin resistance is therefore essential to inform empirical therapy and support antimicrobial stewardship programmes.

The present study investigated temporal trends in fosfomycin resistance among *K. pneumoniae* isolates causing bloodstream infections (BSIs) in hospitalized adults over a seven-year period. To further characterize fosfomycin-resistant isolates, selected phenotypic and molecular analyses, including PPF-based phenotypic assessment of FosA-like enzyme production, polymerase chain reaction (PCR) detection of commonly targeted *fosA* variants, and whole-genome sequencing of representative isolates, were also performed.

## 2. Results

### 2.1. Study Population and Temporal Trends in Fosfomycin Resistance

A total of 198 non-duplicate *K. pneumoniae* bloodstream isolates recovered from hospitalized adults between 2017 and 2023 were included in the study. The demographic and microbiological characteristics of the study population are summarized in Table 1. Overall, 109 (55.1%) isolates were carbapenem-resistant and 50 (25.3%) were classified as fosfomycin-resistant according to the 2023 EUCAST intravenous clinical breakpoint.

According to the 2023 EUCAST intravenous clinical breakpoint, the annual fosfomycin resistance rate increased from 10.3% in 2017 to 45.5% in 2022, although a lower resistance rate was observed in 2023 (21.9%). Binomial logistic regression demonstrated that the odds of fosfomycin resistance increased by 22% for each calendar year (OR 1.22, 95% CI 1.04–1.44; *p* = 0.019) (Figure 1).

When the isolates were re-evaluated using the FDA susceptibility test interpretive criteria (STIC) for intravenous fosfomycin and *K. pneumoniae*, 148/198 (74.7%) isolates were classified as susceptible, 25/198 (12.6%) as intermediate, and 25/198 (12.6%) as resistant. FDA-defined resistance increased from 0% in 2017 to 27.3% in 2022 and was 18.8% in 2023. Logistic regression demonstrated a significant temporal increase in the odds of FDA-defined resistance (OR per year, 1.43; 95% CI, 1.13–1.81; *p* = 0.003).

Using the current EUCAST fosfomycin (T)ECOFF for *K. pneumoniae* (128 mg/L), 12/198 (6.1%) isolates were categorized as non-wild type (non-WT; MIC > 128 mg/L). No non-WT isolates were detected in 2017 or 2018, whereas annual non-WT proportions ranged from 2.9% to 15.4% during 2019–2023. Logistic regression showed an increasing temporal trend in the odds of a non-WT phenotype, although this did not reach statistical significance (OR per year, 1.36; 95% CI, 0.99–1.86; *p* = 0.057).

### 2.2. Comparison According to Carbapenem Resistance and ICU Admission

Using the 2023 EUCAST intravenous clinical breakpoint classification, fosfomycin resistance was significantly more frequent among carbapenem-resistant than carbapenem-susceptible isolates (36.7% vs. 11.2%; OR 4.55, 95% CI 2.05–10.98; *p* < 0.001). Although resistance tended to be more common among ICU isolates, this difference did not reach statistical significance (OR 1.97, 95% CI 0.93–4.13; *p* = 0.066).

### 2.3. Fosfomycin MIC Distribution and Annual Fosfomycin Consumption

Median fosfomycin MIC values did not differ significantly across study years (Kruskal–Wallis test, *p* = 0.721). Annual intravenous fosfomycin consumption showed a positive ecological correlation with fosfomycin resistance defined according to the 2023 EUCAST intravenous clinical breakpoint (Spearman’s ρ = 0.857, *p* = 0.014). Given that this analysis was based on annual aggregated data, the findings should be interpreted as exploratory rather than evidence of a causal relationship.

### 2.4. Phenotypic and Molecular Characterization of Fosfomycin-Resistant Isolates

Among the 47 viable isolates classified as fosfomycin-resistant according to the 2023 EUCAST intravenous clinical breakpoint, 17 (36.2%) were positive in the PPF disk enhancement test (Figure 2). Carbapenem resistance was detected in 37 (78.7%) isolates. Based on the BD Phoenix CPO Detect panel, one isolate (2.1%) was classified as class A, four (8.5%) as class B, and six (12.8%) as class D carbapenemase producers. PPF positivity was not associated with carbapenem resistance (82.4% vs. 76.7%, Fisher’s exact test, *p* = 0.727), carbapenemase class (class A, *p* = 1.000; class B, *p* = 0.128; class D, *p* = 0.653), or fosfomycin MIC values. The median fosfomycin MIC was 128 mg/L among PPF-negative isolates and 64 mg/L among PPF-positive isolates, with no statistically significant difference (Wilcoxon rank-sum test, *p* = 0.225). Likewise, the proportion of PPF-positive isolates did not differ significantly across study years (Fisher’s exact test, *p* = 0.194).

### 2.5. Molecular Characterization of Fosfomycin Resistance

To investigate acquired *fosA* variants, PCR amplification targeting *fosA*, *fosA2*, *fosA3*, and *fosA4* was initially performed using published primer sequences. No amplification was observed for *fosA*, *fosA2*, or *fosA3*. Amplification with the published *fosA4* primers was observed in 33/47 (70.2%) isolates.

Two of these *fosA4* primer-derived amplicons (isolates 25 and 26) were subjected to Sanger sequencing. BLASTx analysis using the NCBI web-based BLAST interface against the NCBI non-redundant protein database showed that the amplified sequence corresponded to the formate dehydrogenase subunit alpha gene (GenBank accession HBV3655157.1), rather than FosA4, demonstrating non-specific amplification by the published *fosA4* primers. A newly designed *K. pneumoniae*-specific *fosA4* primer pair (n*fosA4*) was subsequently tested, and no amplification was observed in any of the isolates.

Two fosfomycin-resistant isolates (isolates 25 and 45) underwent WGS. The circularized chromosome of isolate 25 was 5,346,436 bp in length. Additionally, four circular plasmids of lengths plasmid 1 (64,012 bp), plasmid 2 (62,505 bp), plasmid 3 (5010 bp), and plasmid 4 (3511 bp) were identified. The circularized chromosome of isolate 45 was 5,320,339 bp in length. Additionally, four circular plasmids of lengths plasmid 1 (71,254 bp), plasmid 2 (50,907 bp), plasmid 3 (5010 bp), and plasmid 4 (3511 bp) were identified. Antimicrobial resistance genes were identified in the WGS assemblies using AMRFinderPlus. The *fosA4* gene was not identified in either isolate. In both sequenced isolates, the same predicted chromosomal gene was identified as *fosA5* by AMRFinderPlus.

Exploratory BLASTp comparison of proteins implicated in fosfomycin susceptibility with the corresponding proteins of *K. pneumoniae* ATCC 700721 showed that MurA, GlpT, CyaA, and Crp were identical to the reference proteins in both isolates. Both isolates showed the same amino acid substitutions in UhpT (A462T), UhpA (E69D), and PtsI (N174K). UhpB showed a seven-amino-acid N-terminal length difference together with L8M, M49I, T140A, and Q434H substitutions in both isolates. The whole-genome sequences have been deposited in the NCBI GenBank database under BioProject accession numbers PRJNA1297596 (isolate 25) and PRJNA1303056 (isolate 45).

## 3. Discussion

This seven-year surveillance study demonstrated a significant increase in fosfomycin resistance among *K. pneumoniae* bloodstream isolates from hospitalized adults when MICs were interpreted using the 2023 EUCAST intravenous clinical breakpoint. The annual increase remained significant in logistic regression analysis, corresponding to a 22% increase in the odds of resistance per calendar year. Importantly, reanalysis using the FDA STIC for intravenous fosfomycin and *K. pneumoniae* also demonstrated a significant temporal increase in resistance, with a 43% increase in the odds of resistance per calendar year. In contrast, application of the current EUCAST (T)ECOFF identified a smaller non-WT population, and the temporal increase in the non-WT phenotype did not reach statistical significance. In addition, fosfomycin resistance was significantly more frequent among carbapenem-resistant than among carbapenem-susceptible isolates when assessed using the 2023 EUCAST intravenous clinical breakpoint. These findings highlight the importance of continuous local surveillance to support antimicrobial stewardship as intravenous fosfomycin is increasingly incorporated into the treatment of multidrug-resistant Gram-negative infections.

Intravenous fosfomycin has re-emerged as an important therapeutic option for severe infections caused by multidrug-resistant Gram-negative pathogens, particularly when used in combination regimens [9]. Previous studies have demonstrated good *in vitro* activity against carbapenem-resistant Enterobacterales, with susceptibility rates approaching 80% in some collections [10]. In addition, intravenous fosfomycin was introduced at our institution in late 2018. Although our ecological analysis cannot establish causality, the increasing use of fosfomycin together with the observed increase in resistance underscores the importance of antimicrobial stewardship and continuous local surveillance.

Using the 2023 EUCAST intravenous clinical breakpoint, the overall fosfomycin resistance rate was 25.3%. This rate was comparable to previous reports from Türkiye, although direct comparisons should be interpreted cautiously because of differences in study populations, susceptibility testing methods, and interpretive criteria. Zarakolu et al. reported fosfomycin susceptibility rates of 90.7% among carbapenem-susceptible and 69.4% among carbapenem-resistant *K. pneumoniae* bloodstream isolates using the agar dilution method [11]. Similarly, Demirci-Duarte et al. reported fosfomycin resistance in 46.8% of carbapenemase-producing *Klebsiella* spp. [12]. In our previous study conducted at the same institution, fosfomycin resistance reached 52.7% among carbapenem-resistant *K. pneumoniae* bloodstream isolates collected between 2020 and 2022 [13]. Notably, the highest resistance rates in the present seven-year surveillance study were also observed during the same period, indicating that the high resistance rates observed in our previous study likely reflected a sustained increase in fosfomycin resistance rather than a transient phenomenon.

The different results obtained using the FDA STIC and EUCAST (T)ECOFF emphasize the importance of the interpretive framework applied to fosfomycin MICs in *K. pneumoniae*. Using the FDA STIC, 12.6% of isolates were classified as resistant, and the temporal increase remained statistically significant (OR per year, 1.43; 95% CI, 1.13–1.81; *p* = 0.003). In contrast, only 6.1% of isolates were categorized as non-WT according to the EUCAST (T)ECOFF, and the temporal trend did not reach statistical significance (OR per year, 1.36; 95% CI, 0.99–1.86; *p* = 0.057). This difference reflects the distinct purposes of clinical breakpoints and epidemiological cutoffs: clinical breakpoints are intended to support categorization of antimicrobial susceptibility in a therapeutic context, whereas ECOFFs distinguish organisms with acquired or mutational resistance mechanisms from the phenotypically wild-type population. Accordingly, clinical susceptibility categories and WT/non-WT classification should not be interpreted as interchangeable.

The period during which fosfomycin resistance peaked in our study coincided with the COVID-19 pandemic. Several studies have reported increased antimicrobial consumption and rising rates of antimicrobial resistance during this period, likely reflecting changes in antibiotic prescribing practices and prolonged hospitalization of critically ill patients [14]. Although our study was not designed to evaluate the impact of the pandemic, this temporal overlap may have contributed to the observed resistance trend.

Despite the increase in resistance rates over time, median fosfomycin MIC values remained stable throughout the study period, suggesting that the observed trend reflected an increasing proportion of resistant isolates rather than progressively higher resistance levels among resistant strains.

Fosfomycin resistance in *K. pneumoniae* is mediated by multiple mechanisms, including alterations in the target enzyme MurA, impaired fosfomycin uptake through the GlpT and UhpT transport systems and their regulatory genes, and FosA enzymes that enzymatically inactivate fosfomycin [2,6,15]. Plasmid-mediated *fosA* variants are of particular epidemiological importance because of their potential for horizontal dissemination, whereas chromosomal *fosA* homologues are considered intrinsic to *K. pneumoniae* [16,17,18]. The targeted PCR component of the present study therefore focused on acquired *fosA* variants because of their potential for horizontal dissemination and epidemiological relevance. This approach, however, was not intended to comprehensively characterize all mechanisms of fosfomycin resistance. The commonly targeted acquired *fosA* variants (*fosA*, *fosA2*, *fosA3*, and *fosA4*) were not detected. Although amplification was initially observed with the published *fosA4* primers, Sanger sequencing demonstrated that these products represented non-specific amplification of the formate dehydrogenase subunit alpha gene rather than *fosA4*. Subsequent testing with newly designed *K. pneumoniae*-specific *fosA4* primers yielded no amplification. These findings highlight the importance of validating published PCR assays before their application to *K. pneumoniae* isolates.

Whole-genome sequencing of two representative isolates identified the chromosomal *fosA5* locus. Exploratory comparison of selected proteins involved in fosfomycin susceptibility showed no amino acid differences in MurA, GlpT, CyaA, or Crp relative to *K. pneumoniae* ATCC 700721. Both isolates showed UhpT A462T, UhpA E69D, and PtsI N174K substitutions, while UhpB showed a seven-amino-acid N-terminal length difference together with L8M, M49I, T140A, and Q434H substitutions. Notably, UhpT A462T, PtsI N174K, and UhpB T140A have previously been reported in *K. pneumoniae* isolates with variable fosfomycin MICs [2], indicating that their presence alone should not be considered sufficient evidence of a resistance mechanism. The functional significance of the other observed sequence differences remains uncertain. Because only two isolates underwent genomic analysis and sequencing was based exclusively on Nanopore long reads, these exploratory findings should be interpreted cautiously and cannot be generalized to the entire collection or used to establish a causal mechanism of fosfomycin resistance.

PPF disk enhancement testing has been proposed as a simple phenotypic method for detecting FosA-mediated fosfomycin resistance in clinical laboratories [4,8]. Guo et al. demonstrated that PPF substantially reduced fosfomycin MICs in *K. pneumoniae* and *E. coli* isolates carrying chromosomal or plasmid-mediated *fosA* genes [19]. In the present study, 36.2% of fosfomycin-resistant isolates were PPF-positive. However, PPF positivity was not associated with carbapenem resistance, carbapenemase class, fosfomycin MIC values, or the year of isolation. Because the commonly targeted acquired *fosA* variants were not detected and only two isolates underwent whole-genome sequencing, the molecular basis of PPF positivity could not be determined. Further studies combining phenotypic PPF testing with comprehensive genomic characterization are needed to clarify the diagnostic value of this assay in *K. pneumoniae*.

This study has several limitations. First, whole-genome sequencing was performed for only two representative isolates; therefore, the molecular findings cannot be generalized to the entire collection of fosfomycin-resistant isolates. Furthermore, sequencing was performed using Oxford Nanopore technology without complementary short-read sequencing, which may limit the accuracy of small-variant detection. Second, the targeted PCR analysis was restricted to selected acquired *fosA* variants and therefore could not comprehensively determine the molecular mechanisms underlying fosfomycin resistance across the collection. Chromosomal mechanisms of fosfomycin resistance were explored only in the two sequenced isolates and were not systematically investigated across the entire collection. Therefore, the contribution of alterations in *murA*, *glpT*, *uhpT*, *uhpA*, *uhpB*, *cyaA*, *ptsI*, and *crp* to fosfomycin resistance in the overall study population could not be determined. Third, the correlation between annual intravenous fosfomycin consumption and resistance rates was based on aggregated annual data and should be interpreted as an exploratory ecological observation rather than evidence of a causal relationship. Finally, this was a single-center study, and resistance trends may differ across institutions with different patient populations and antimicrobial prescribing practices.

## 4. Methods

### 4.1. Study Design, Bacterial Isolates and Antimicrobial Susceptibility Testing

This research was carried out at a university hospital with a capacity of 900 beds, recognized as the largest facility in the Southern Marmara region of Türkiye. A total of 198 non-duplicate *K. pneumoniae* isolates recovered from blood cultures of hospitalized adults between 2017 and 2023 were included. Only adult inpatients were included in the study, and each patient was represented by a single isolate. Annual intravenous fosfomycin consumption (g/year) was obtained from the hospital pharmacy records.

The isolates were subcultured on 5% sheep blood agar plates (Becton Dickinson GmbH, Heidelberg, Germany) and incubated at 37 °C overnight. Identification was performed using matrix-assisted laser desorption ionization time-of-flight mass spectrometry (MALDI-TOF MS) (Bruker Daltonik, Bremen, Germany) with the IVD v 12.0 database. Only isolates with a score of two or higher were included in the subsequent *in vitro* antimicrobial susceptibility tests.

Fosfomycin susceptibility was determined by the agar dilution method following CLSI recommendations [20]. All stored isolates collected between 2017 and 2023 were tested in 2023, and MICs were interpreted using the 2023 EUCAST intravenous fosfomycin clinical breakpoint (susceptible, ≤32 mg/L; resistant, >32 mg/L) [21]. The same breakpoint was applied to all isolates irrespective of the year of isolation. MICs were additionally evaluated using the FDA-identified susceptibility test interpretive criteria (STIC) for intravenous fosfomycin and *K. pneumoniae* (susceptible, ≤32 mg/L; intermediate, 64 mg/L; resistant, ≥128 mg/L) [22]. As an epidemiological analysis, MICs were also evaluated using the current EUCAST fosfomycin (T)ECOFF for *K. pneumoniae* (128 mg/L), with isolates categorized as wild type (WT; MIC ≤ 128 mg/L) or non-wild type (non-WT; MIC > 128 mg/L) [23]. For AD susceptibility testing, we used cation-adjusted Mueller–Hinton Agar (CAMHA) with 25 µg/mL of glucose-6-phosphate (CAMHA-G6P). The antibiotic media were prepared in a water bath at 40–50 °C, and doubling concentrations of fosfomycin were incorporated into CAMHA supplemented with 25 mg/L glucose-6-phosphate [24]. In the AD method, a bacterial suspension of each test organism was prepared at a concentration of 10^7^ CFU/mL (1/10 McFarland 0.5), and 2 μL of bacteria was inoculated onto CAMHA-G6P plates with varying concentrations of fosfomycin. The plates were incubated for 18–20 h at 37 °C. Experiments were conducted at least three times on different days, with *E. coli* ATCC 25922 used as a quality control strain. Isolates were stored at −20 °C. The MIC range for fosfomycin was determined according to CLSI recommendations, with fosfomycin concentrations tested ranging from 0.125 to 128 mg/L. Fosfomycin disodium salt (Sigma-Aldrich, St. Louis, MO, USA), glucose-6-phosphate (G6P) disodium salt (Biobasic, Markham, ON, Canada) and PPF (Sigma-Aldrich, St. Louis, MO, USA) were sourced commercially. Antibiotic susceptibility testing for all other antibiotics was conducted using a commercial system, (BD Phoenix M50 System, Sparks, MD, USA) in accordance with the 2023 EUCAST guidelines [21]. We conducted susceptibility testing for carbapenem antibiotics (meropenem, imipenem, and ertapenem) in the same isolates using an automated commercial system.

For further phenotypic and molecular characterization, fosfomycin-resistant isolates were recovered from frozen stocks. Fifty fosfomycin-resistant isolates were cultured on blood agar to investigate the presence of *fosA* genes. Isolates were numbered from one to 50. Three isolates (numbers 5, 13, and 48) could not be recovered after repeated subculture attempts from frozen stocks, including inoculation onto sheep blood agar, thioglycollate broth, and blood culture bottles, and were therefore excluded from subsequent analyses.

### 4.2. Phenotypic and Molecular Characterization of Fosfomycin-Resistant Isolates

DNA isolation was performed using the PureLink™ Genomic DNA Mini Kit (Invitrogen, Carlsbad, CA, USA) according to the manufacturer’s instructions. DNA extracted from the 47 isolates was stored at −20 °C until required.

Polymerase chain reaction amplification was performed in a 25 µL reaction volume consisting of NEB OneTaq Hot Start Quick-Load 2X Master Mix (New England Biolabs, Ipswich, MA, USA), 0.5 µM of each primer, and 50–100 ng of template DNA. Thermal cycling for *fosA* primers included a pre-denaturation step at 94 °C for 30 s, followed by 35 cycles of denaturation at 94 °C for 30 s, annealing at 55 °C for 30 s, and extension at 68 °C for 30 s, with a final extension at 68 °C for 5 min. The other annealing temperatures are given in Table 2. PCR products were separated by 2% agarose gel electrophoresis.

PCR products selected for sequence verification were purified and subjected to unidirectional Sanger sequencing at BM Labosis Biotechnology Laboratory (Ankara, Türkiye). Sequencing was performed on an ABI PRISM^®^ 3730xl DNA Analyzer (Applied Biosystems, Foster City, CA, USA) using BigDye™ Terminator v3.1 Cycle Sequencing chemistry according to the manufacturer’s instructions.

Because previously published *fosA4* primers produced unexpected amplification products, a new *K. pneumoniae*-specific primer set (n*fosA4*) was designed using the genome sequence of *K. pneumoniae* strain 201412878M (GenBank accession DAJDDX010000051.1). Primers were designed using the BV-BRC Primer Design Pipeline (Primer3) [25,26] and evaluated in silico with Primer-BLAST to confirm specificity [27]. Primer sequences [28,29,30] are shown in Table 2.

**Table 2:** Oligonucleotide primers utilized in our study for the detection of fosfomycin resistance genes.

Target Gene	Sequence(5′ → 3′)	Amplicon Size, bp	Annealing Temperature	Reference
*fosA*	F: ATCTGTGGGTCTGCCTGTCGT R: ATGCCCGCATAGGGCTTCT	271	55 °C	[28,29,30]
*fosA2*	F: GCTGCAATCACTCAACCATC R: CACGTGCAGCTCCAGCTT	346	54 °C	[28,29,30]
*fosA3*	F: GCGTCAAGCCTGGCATTT R: GCCGTCAGGGCTGAGAAA	282	54 °C	[28,29,30]
*fosA4* (first primers)	F: CTGGCGTTTTATCAGCGGTT R: CTTCGCTGCGGTTGTCTTT	230	52 °C	[28,29,30]
n*fosA4* (redesign primers)	F: AGCATATCTTTCCTGCGGGG R: GCCAAATGACCCACATGCAG	249	54 °C	in house, this study

F, forward; R, reverse.

Two representative isolates with high fosfomycin MICs (isolates 25 and 45) were selected for exploratory whole-genome sequencing (WGS) using the Oxford Nanopore PromethION platform (Oxford Nanopore Technologies, Oxford, UK). Sequencing libraries were prepared from genomic DNA with the Oxford Nanopore ligation kit (SQK-NBD114-24; Oxford Nanopore Technologies, Oxford, UK) and were run on the P2 Solo sequencer for 24 h. The ONT data obtained in FAST5 format from the sequencing were converted to high-fidelity (HAC) FASTQ files using the Guppy basecaller (v6.1.7). These FASTQ files were then uploaded to the BV-BRC (Bacterial and Viral Bioinformatics Resource Center) server, where quality control and normalization were performed using BBNorm (v38.90). Assemblies were completed using the Autocycler pipeline (v0.5.0). Filtered reads were subsampled and assembled using Flye (v2.9.6-b1802), Miniasm (v0.3.r179), and Raven (v1.8.3). Generated contigs were clustered and reconciled, and consensus sequences were generated. Consensus sequences were polished with Medaka (v1.11.3) using long-reads. The draft genome sequences were annotated by Prokka (v1.14.6) using the annotated genome sequence of *K. pneumoniae* subsp. *pneumoniae* HS11286 as a reference (PRJNA78789, NCBI BioSample accession SAMN02602959). Antibiotic resistance gene analysis was conducted using AMRFinderPlus (v4.0.19).

As an exploratory analysis of chromosomal mechanisms potentially associated with fosfomycin susceptibility, the predicted amino acid sequences of MurA, GlpT, UhpT, UhpA, UhpB, CyaA, PtsI, and Crp from both sequenced isolates were compared with the corresponding proteins of *K. pneumoniae* ATCC 700721, following the reference-based approach described by Ortiz-Padilla et al. [2]. Protein sequences were compared using NCBI BLASTp to identify amino acid substitutions and major length differences.

Phenotypic evidence of FosA-like enzyme production was evaluated using a sodium phosphonoformate (PPF)-based disk potentiation test as previously described by Elliott et al. [4]. Briefly, bacterial suspensions equivalent to a 0.5 McFarland standard were inoculated onto cation-adjusted Mueller–Hinton agar supplemented with 25 mg/L glucose-6-phosphate (CAMHA-G6P). Two 200-µg fosfomycin disks and one blank disk were placed on each plate. A 20-µL aliquot of freshly prepared PPF solution (50 mg/mL) was applied to one fosfomycin disk and the blank disk. Following overnight incubation at 37 °C, inhibition zone diameters were measured. An increase of ≥5 mm in the inhibition zone around the PPF-containing fosfomycin disk, compared with the fosfomycin disk alone, was interpreted as phenotypic evidence of FosA-like enzyme production.

### 4.3. Statistical Analysis

Statistical analyses were performed using R version 4.4.2 (R Foundation for Statistical Computing, Vienna, Austria). Categorical variables are presented as frequencies and percentages and continuous variables as mean ± standard deviation or median with interquartile range, as appropriate. Annual fosfomycin resistance rates were calculated with 95% Wilson confidence intervals. Temporal trends in fosfomycin resistance were evaluated using binomial logistic regression with calendar year as a continuous predictor. Separate logistic regression analyses were performed for resistance defined according to the 2023 EUCAST intravenous clinical breakpoint, resistance defined according to the FDA susceptibility test interpretive criteria (STIC), and the non-wild-type (non-WT) phenotype defined according to the EUCAST (T)ECOFF. Associations between categorical variables were assessed using the chi-square test or Fisher’s exact test, as appropriate. Fosfomycin MIC distributions across study years were compared using the Kruskal–Wallis test, and MIC values between PPF-positive and PPF-negative isolates were compared using the Wilcoxon rank-sum test. The relationship between annual intravenous fosfomycin consumption and annual resistance rates defined according to the 2023 EUCAST intravenous clinical breakpoint was evaluated using Spearman’s rank correlation as an exploratory ecological analysis. Figures were generated using the ggplot2 package in R. A two-sided *p* value < 0.05 was considered statistically significant.

## 5. Conclusions

In conclusion, this seven-year surveillance study demonstrated an increasing temporal trend in fosfomycin resistance among *K. pneumoniae* bloodstream isolates from hospitalized adults, with a significant increase observed using both the 2023 EUCAST intravenous clinical breakpoint and the FDA susceptibility test interpretive criteria. In contrast, the increase in the non-WT phenotype based on the current EUCAST (T)ECOFF did not reach statistical significance. The observed ecological association between annual intravenous fosfomycin consumption and resistance rates highlights the importance of continuous surveillance and antimicrobial stewardship, although a causal relationship cannot be inferred. The absence of the commonly targeted acquired *fosA* variants and the non-specific amplification observed with previously published *fosA4* primers emphasize the need for careful molecular validation when investigating fosfomycin resistance. Exploratory genomic analysis of two representative isolates identified chromosomal *fosA5* and sequence differences in selected proteins involved in fosfomycin susceptibility; however, their contribution to resistance could not be established. Further genomic studies are warranted to clarify the contribution of chromosomal resistance mechanisms to fosfomycin resistance and to support future antimicrobial stewardship efforts.

## Figures and Tables

**Figure 1:**
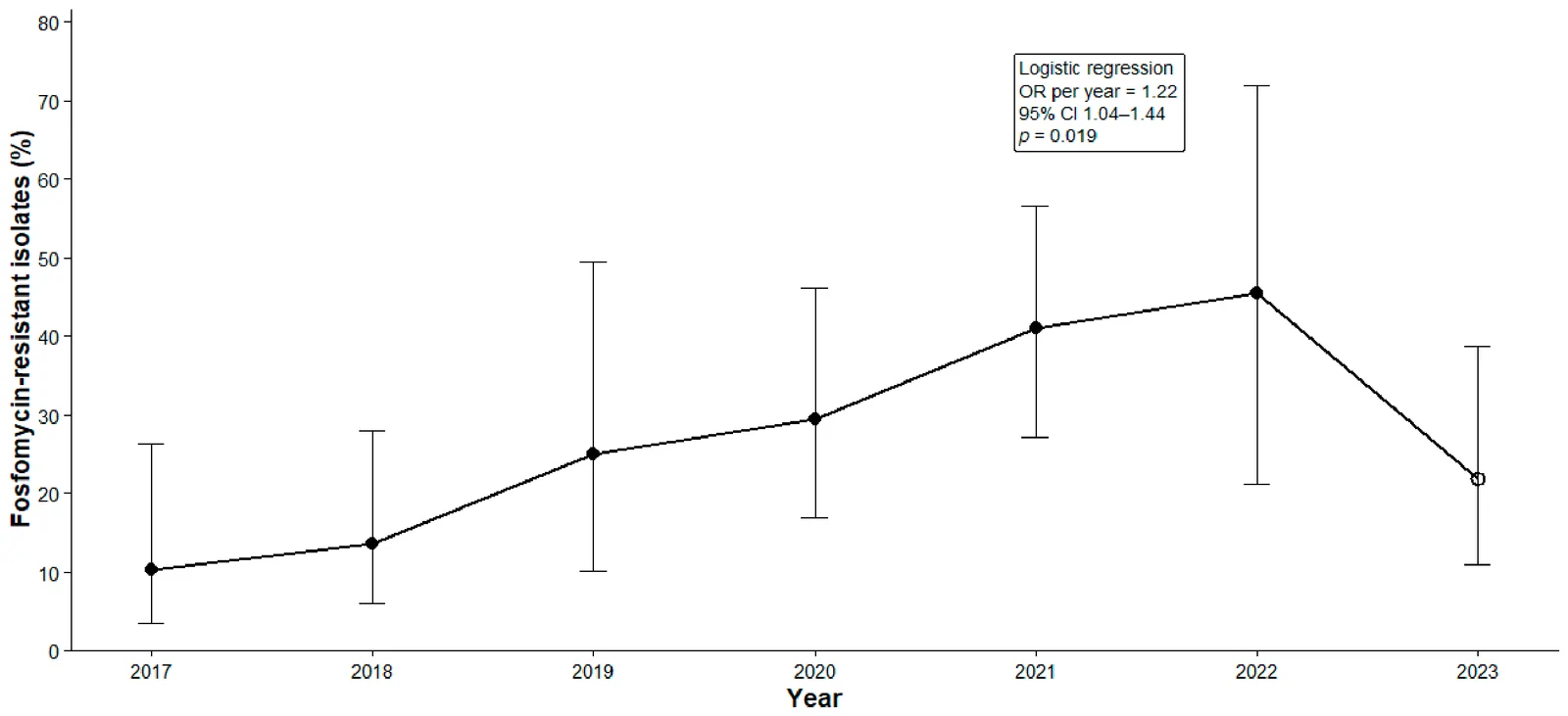
Annual fosfomycin resistance rates among *Klebsiella pneumoniae* bloodstream isolates according to the 2023 EUCAST intravenous clinical breakpoint. Error bars indicate 95% Wilson confidence intervals.

**Figure 2:**
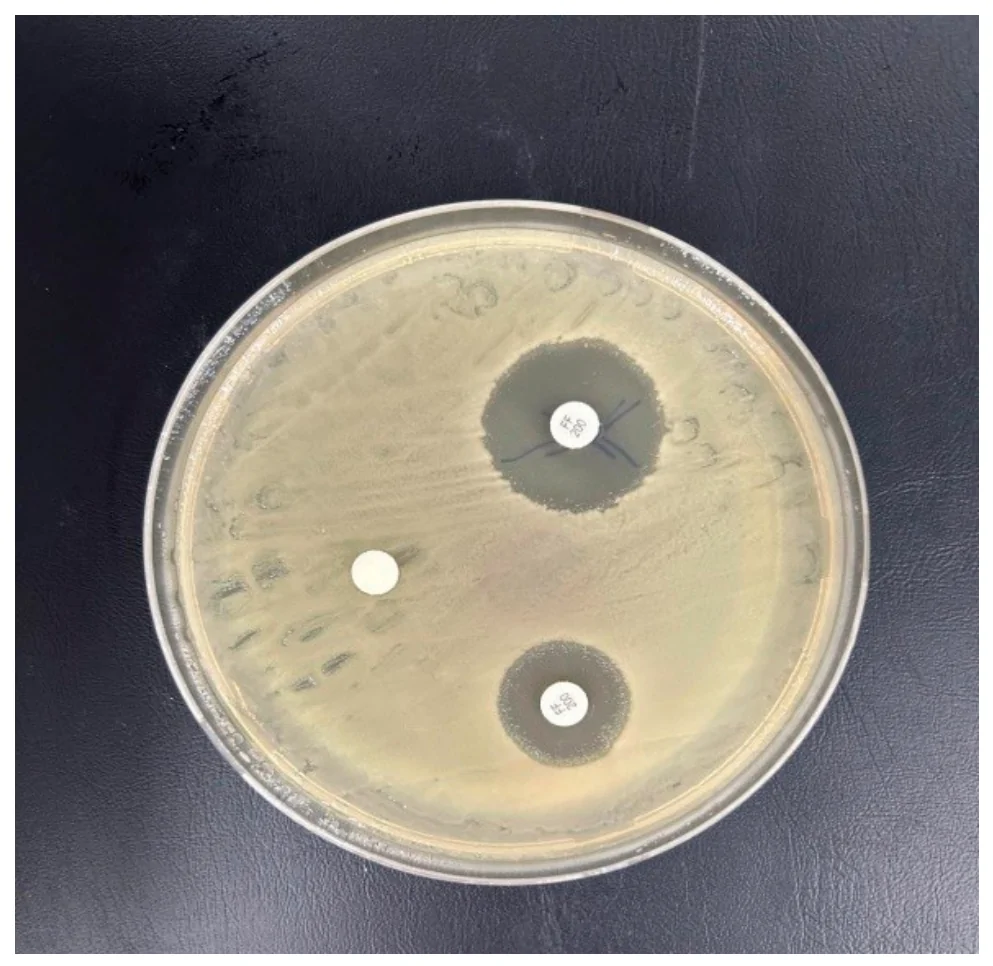
Representative PPF-based disk potentiation test demonstrating an increased fosfomycin inhibition zone in the presence of PPF.

**Table 1:** Baseline characteristics of hospitalized adults and *Klebsiella pneumoniae* bloodstream isolates included in the study.

Characteristic	Value
Number of isolates	198
Age, years, mean ± SD	57.1 ± 16.6
Male sex	126 (63.6%)
ICU	54 (27.3%)
Carbapenem-resistant isolates	109 (55.1%)
Fosfomycin-resistant isolates according to the 2023 EUCAST intravenous clinical breakpoint	50 (25.3%)

## Data Availability

The whole-genome sequencing data have been deposited in the NCBI GenBank database under BioProject accession numbers PRJNA1297596 (isolate 25; GenBank accession JBPZNI000000000; BioSample SAMN50228938) and PRJNA1303056 (isolate 45; GenBank accession JBXWUN000000000; BioSample SAMN50493735).
